# Assessing the effects of online educational intervention on pharmaceutical promotions: a pre–post study among medical and pharmacy students in Pakistan

**DOI:** 10.3389/fphar.2025.1616631

**Published:** 2025-08-04

**Authors:** Ali Hassan Gillani, Hafsa Arshad, Caijun Yang, Muhammad Arshed, Naveel Atif, Kamran Bashir, Hafiz Muhammad Abbas Malik, Yu Fang

**Affiliations:** ^1^ Department of Pharmacy Administration and Clinical Pharmacy, School of Pharmacy Xi’an Jiaotong University, Xi’an, Shaanxi, China; ^2^ Center for Drug Safety and Policy Research, Xian Jiaotong University, Xi’an, Shaanxi, China; ^3^ Shaanxi Center for Health Reform and Development Research, Xi’an, Shaanxi, China; ^4^ Department of Community Medicine, Baqai Medical College, Baqai Medical University, Karachi, Sindh, Pakistan; ^5^ College of Pharmacy, University of Sargodha, Sargodha, Pakistan; ^6^ Department of Pharmacy, The Islamia University of Bahawalpur, Bahawalpur, Punjab, Pakistan

**Keywords:** online, educational intervention, pharmaceutical promotion, medical students, pharmacy students, Pakistan

## Abstract

**Background:**

Pharmaceutical promotion tend to alter prescribing and dispensing behaviors pharma companies target undergraduate students. Teaching about such promotion is inadequate, and little research has been done in Pakistan regarding this issue. Therefore, we designed and implemented an online educational intervention on pharmaceutical promotion and evaluated its effect on the knowledge and attitude of medical and pharmacy students.

**Methods:**

An online interventional study was conducted among pharmacy and medical students to evaluate their pre and post perception and attitude score using a questionnaire. The module was developed by information from the WHO/HAI module on pharmaceutical promotion. The pre–post median score was calculated for each item. Demographic variation in median score was evaluated by Mann–Whitney and Kruskal–Wallis tests, and total median scores before and after the module were compared using the Wilcoxon signed ranks test. We analyzed the data by using SPSS version 23.0 for Windows.

**Results:**

There is significant modification in most of the perception and attitude items of pharmacy and medical students (p < 0.001). There is also a significant decrease in the total perception, attitude, and median scores of the medical and pharmacy students (p < 0.001). In terms of demographic variations, female students tend to show lower perception and attitude scores in both the cohort. Medical students from private organizations showed lower pre and post scores than those from government medical schools.

**Conclusion:**

There is a significant decrease in inclination toward promotion after the module in medical and pharmacy students. It shows the effectiveness of online intervention provided to students. There should be teaching for healthcare students on how to choose and use independent information sources to assess pharmaceutical promotions.

## Introduction

According to the World Health Organization (WHO), pharmaceutical promotion (PP) Describe as “…all information and persuasive activities by manufacturers, the effect of which is to induce the prescription, supply, purchase and/or use of medicinal drugs” (World Health Organization, 2003). These tactics encompass a wide variety of events, such as the provision of gifts, free medication samples, printed brand materials, and sponsored conferences, lunches, and tickets (Jacob, 2018). Healthcare students will ultimately acquire employment in the future, and their decisions can bring change in the community. These decisions are largely affected by educational experiences through teaching and training (Gillani et al., 2024a; Gul et al., 2021). Pharmaceutical companies (PCs) evaluate the implementation of promotional activities for undergraduate students, and their influence starts at this level (Gillani et al., 2024a; Gul et al., 2021; Gillani et al., 2022a; Gillani et al., 2022b; Ball and Al-Menea, 2018). Changes in drug handling practices, changes of behavior, irrationality, and obligation to PCs result from these promotional activities (Gillani et al., 2024a; Mandiracioglu and Kiran, 2014; Gillani et al., 2024b).

Students who receive promotional items or gifts from PCs show stronger product affinities because these items create brand commitment (Gillani et al., 2024a; Mandiracioglu and Kiran, 2014; Gillani et al., 2024b; Ingole and Yegnanarayan, 2011; Austad et al., 2011; Shankar et al., 2012). Students request education about PP (Mandiracioglu and Kiran, 2014; Gillani et al., 2024b; Ingole and Yegnanarayan, 2011; Austad et al., 2011), so it is the utmost duty of medical educators and mentors to teach students how to understand and use information from medical representatives (MRs) (Shankar et al., 2012). Students showed little to no interest in learning about PP if schools force them to study it as a compulsory subject or assign it to them as excessive workload (Shankar et al., 2012). A previous worldwide survey of medical and pharmacy schools which incorporated PP education into their curriculum revealed that students generally dedicated only 1 or 2 hours or a total of less than 1 day to this essential subject during their course curriculum (Shankar et al., 2012; Mintzes, 2005). A comprehensive review (Norris et al., 2005) indicated that self-regulation by the pharmaceutical industry (PI), journal editor oversight, marketing representative advertising guidelines, and post-marketing rules fail to reduce promotional problems caused by PCs. Official government regulations together with research-based deception identification and medical prescriber education about promotional methods are effective strategies to control the effects of PP (Norris et al., 2005; Civaner, 2020).

Drug promotion can act as a differentiating medium between a drug being a “poison” or a “cure” (Leonardo Alves et al., 2019; Lexchin, 2012; Ziganshina et al., 2009). Pakistan’s *National Code of Pharmaceutical Marketing Practices* exists but lacks enforcement. Unqualified “doctors” receive drug promotions, and transparency mechanisms are absent (RAHEEM and TARIQ, 2010). Physician prescription practices are being significantly impacted by a massive push of drug promotion, which is causing doctors to prescribe drugs irrationally (Diekema, 2022; Kasse et al., 2024; Machowska and Stålsby Lundborg, 2019). This practice has been common in Pakistan (Noor et al., 2023), and evidence shows that the practices of pharmacists (Gillani et al., 2022b) and views of the public (Gillani et al., 2022a), medical (Gillani et al., 2024a), and pharmacy students (Gillani et al., 2024b) have been greatly influenced by promotional tactics to ultimately increase irrationality around drugs and antibiotics. PP must be studied not merely as a business tactic but as a determinant of patient outcomes. In Pakistan, unregulated practices exacerbate antibiotic resistance and healthcare costs. By integrating promotion literacy into education, enforcing transparency, and decoupling professional development from industry funding, prescribers can realign with evidence-based care. As frontline advocates, physicians and pharmacists must lead this reform—recognizing, as one critic warns, that “…patients suffer when profit eclipses professionalism” (Mishra, 2025). However, no study is apparent which evaluates the effect of intervention or training on students in Pakistan. It is thus necessary to test the impact of an online module on students’ knowledge, attitudes, and practices regarding PP in Pakistan. Therefore, we decided to test the impact of an online educational intervention on such knowledge, attitude, and practices using a retrospective pre–post online questionnaire.

## Materials and methods

### Study area, study population, and design

Pakistan consists of the provinces of Punjab, Sindh, KPK, and Baluchistan alongside the two Independent Administrative territories of Gilgit Baltistan and Azad Jammu Kashmir and the capital region of Islamabad (Pakistan beurre of statistics. Population census, 2025). The country offers education at six distinct levels, and Pharm-D and MBBS are in graduate (bachelor’s) level degrees (Education in Pakistan, 2025). The Pharm-D degree is awarded by pharmacy colleges regulated by the Higher Education Commission (HEC), and the MBBS degree is awarded by medical colleges regulated by the Pakistan Medical Council (PMC) and HEC (Education in Pakistan, 2025). Pakistan currently operates 176 medical colleges, including 45 public and 72 private institutions, that grant MBBS degrees (Wikipedia, 2025a), and 68 pharmacy Incomplete colleges deal with the Pharm D degree (Wikipedia, 2025b). We chose Punjab as the data collection site because of its large population, extensive healthcare infrastructure, and significant contributions to public health initiatives. As the most populous province, Punjab plays a vital role in achieving national health goals and providing accessible healthcare to a large proportion of the population. It also contains the greatest number of medical and pharmacy colleges in Pakistan. We randomly selected three populous cities of Punjab: Lahore, Rawalpindi and Sialkot.

This research was conducted in two phases. In the beginning of the first phase, we tried to contact professors and students from the government and private medical and pharmacy colleges in Punjab province. As this occurred during COVID-19, a physical approach to universities and medical colleges was limited, so tried to approach the authorities and professors/assistant professors via email and the phone numbers given on the university website. We provided the basic idea and the motives of the research, and we asked to share this with a number of class representatives (CRs) in the third, fourth, and fifth years of their study. Three private and three government medical and pharmacy colleges agreed to participate in the first phase and shared the numbers or contact information for the CRs. The CRs of all three grades were asked to share the online link of the survey instrument with as many students in their classes as they could. We employed snowball sampling, and the survey was made available on Google Forms. All female and male medical undergraduates currently enrolled in the third, fourth, or final year of study in medical or pharmacy colleges were targeted. We opted for an online platform for data acquisition because it was too challenging to conduct paper-based or observational studies during partial or complete lockdown, and a large proportion of people (76 million people) frequently use the internet, with 37 million active users in Pakistan (Gillani et al., 2017) (Anjum, 2020).

In the second phase, the students who participated and completed the baseline survey were given information about the educational intervention and how to join the WhatsApp group. Willing students who then contacted the PI to be part of the interventional program were then added to the WhatsApp group. Separate groups were formed for the medical and pharmacy students. We informed the students of the 1-month window for being part of this study and that willing students should enter the group within the specified period. At the beginning of the baseline survey, every student was asked to select a code/identification number (IN) before completing the questionnaire. Those students who showed intent to be part of the post-survey were then asked to change their number or name in the group by that specific code so as to assure anonymization and identification in the pre–post survey. This IN was also used during the online class Zoom or TenCent meeting. Students who forgot their IN or those whose IN did not match the pre-survey were excluded from the pre–post evaluations. The interventions were done separately on the medical and pharmacy students at different times. The willing students were divided into small groups for the Zoom meeting, not exceeding 50 per group.

### The intervention

We created an educational initiative called the “Pharmaceutical Promotion Interventional Program” (PPIP) based on our comprehensive review of existing literature. The literature review revealed various PP modules and programs, yet our goal to help future physicians and pharmacists develop critical thinking skills (Mansfield, 2019) and an understanding of promotional methods led us to create modules based on national and international guidelines (Civaner, 2012; Civaner et al., 2016). We developed our program using the *Understanding and Responding to Pharmaceutical Promotion* manual published by Health Action International (HAI) and the World Health Organization (WHO) (Mintzes, 2010). This manual functions as an educational resource to support teachers who teach students about PP. The program utilized rules for university–industry relations from developed countries to achieve these goals (Lewis et al., 2001). We gathered all necessary materials before finalizing the educational goals for the upcoming 3-month teaching period.

The main points addressed in the course were as follows.

1) Types of interactions with the PI:
  • rights and responsibilities of different parties
  • related legal regulations and professional codes
2) Nature and motives of a PC as an entity:
  • marketing as a scientific discipline
  • types of promotional methods and how they operate
3) Influential effects of promotional methods on clinical decisions.
4) Soundness of arguments for and against physician–PC interactions
5) Protection from the negative impacts of PC promotion methods
6) Individual measures:
  • rational prescribing/dispensing
  • reaching out to independent scientific information sources
  • avoiding promotional influence

This module was categorized and accepted for all international PP standards and has been well adopted in previous studies (Shankar et al., 2012; Civaner, 2020; Mar et al., 2008). For local regulations, we gathered information from the guidelines given by the Drug Regulatory Authority of Pakistan (DRAP) in “Advertisement of therapeutic good” (Advertisement of Therapeutic Goods, 2022). These guidelines were updated in 2021.^1^

The content drafted from these rules was as follows.

1) Local conditions for the advertisement
2) Prohibitions
3) Penalties
4) Regional limitations and obligations regarding the PP
5) Basic definitions such as MRs and therapeutic goods
6) Type and control of advertisement
7) Ethics and principles for conforming to the contents of the promotion

The above literature was organized as a 6-hour course divided into two 3-hour lectures over 2 weeks. Those who became part of the pre–post evaluations were requested to take both lectures so as to complete the course. Those who took a class on day 1 and missed the other were given another chance to complete the course a few days later. Attendance was taken by monitoring the code numbers in the Zoom meeting groups and engaging with them during the course by calling their IN and asking frequent questions. As the number of students was large, we divided the students into batches and then gave the educational classes. Any students who did not complete the course in the two terms were not considered for the post evaluations. The course was delivered by the principal investigator to all willing students to ensure the same level of training for all individuals. During the course, each class was given a lecture by a professional from a multinational company who was a specialist in marketing education. We employed both classical methods of lectures and interactive group sessions. We also enhanced the understanding of students by showing a short video called “Side Effect” (Slattery-Moschka, 2005) about the professional life of a MR and asked them to spot the promotion methods; ways of implementing those methods were then discussed.

### Tool development and validations

Two different tools were used to evaluate the perceptions: the attitudes and practices of medical and pharmacy students.

For the medical students, an amalgam of previous studies acted as sources for tool development (Ball and Al-Menea, 2018; Siddiqui et al., 2014; Soyk et al., 2010; Sarikaya et al., 2009; Sandberg et al., 1997; Vainiomaki et al., 2004; Fitz et al., 2007; Wilkes and Hoffman, 2001). A comprehensive 45-item instrument was used which was developed based on the above literature. The questionnaire sought information in four main sections: Section 1: demographics of medical students (4 items); Section 2: perception of medical undergrads about PP (10 items); Section 3: attitudes/behaviors of medical students toward PP (29 items); Section 4: further investigation of the two questions regarding their future practice in terms of the antibiotic prescribing. As there were no legal rules and regulations in our medical setting regarding interactions between PCs and medical students, AMSA guidelines were followed. The detail, content and validation process is given in the baseline studies (Gillani et al., 2024a). In order to make comparisons, the pre-data were evaluated as all items in parts 2 (Perception) and 3 (Attitude) on 5-point Likert scales (5 = strongly agree, 4 = agree, 3 = neutral, 2 = disagree, and 1 = strongly disagree). Cumulative scores were calculated for the perception and attitudes section to describe medical students. Some questions have negative consequences, so the answers “strongly disagree” give the highest score: “strongly disagree” = 5 and “strongly agree” = 1. The collective score of each student was then measured. The cumulative score for attitude ranges 10–50, for perception 29–145, and 2–10 for practices.

The research tool for pharmacy students combined 47 items obtained from various studies involving pharmacy and medical students (Ball and Al-Menea, 2018; Mandiracioglu and Kiran, 2014; Mintzes, 2005; Ashker and Burkiewicz, 2007; Rogers et al., 2004; Bellin et al., 2004). The questionnaire divided its inquiries into four sections: demographic information about pharmacy students (4 items), pharmacy students’ understanding of PP (19 items), their promotional attitudes and behaviors (22 items), and questions about antibiotic practices (2 items). Complete details about the tool, pilot evaluation, and validation study are provided in Gillani et al. (2024b) and Mandiracioglu and Kiran (2014). An overall representation of pharmacy students’ perceptions and attitudes toward PP and PCs was generated by calculating the scores from all perception, attitude, and practice sections. The same above innovative scoring method was also adopted for pharmacy students. The “Perception” section’s scoring spans 19–95 points, “Attitude” scored 22–110 points, and “Practice” 2–10 points. Student scores correlate with their level of affinity toward PP and gifts and PCs but not toward practice where lower scores indicate reduced PC influence on antibiotic prescribing/dispensing.

### Statistical analysis

The data were analyzed using SPSS version 23.0 for Windows. One sample Kolmogorov–Smirnov test was used to determine the normality of the variables. Most variables were found to not follow a normal distribution, and hence non-parametric tests were used. Median scores were compared according to gender, nature of institution, monthly parental income, and years of schooling before and after the module, using a Mann–Whitney test. Median scores before and after the module were compared using the Wilcoxon signed ranks test. A p value less than 0.05 was taken as statistically significant.

### Ethical approval

This interventional study was approved by the Medical Ethics Committees of Xi’an Jiaotong University China and the Ethical Review Board of the University of Lahore. Online pre-approval consent from each student was obtained by clicking “Agree” at the beginning of the online questionnaire. The questionnaire could not commence without agreement with the ethical statement. As the intervention was online and the students had to join WhatsApp groups, their numbers were shown in the groups; this was relayed to them before the commencement of the study; if they agreed, they could be part of the study. Participants were assured that the data would only be used for research purposes and would be treated with confidentiality. We encouraged them to complete the whole study, but participation was voluntary and free of any incentive or penalty, so students could leave the study at any time.

## Results

### Demographic details of medical and pharmacy students

In the baseline study, 1,227 medical students agreed and completed the questionnaire, and 902 students initially agreed to be part of the second phase, although 36 failed to complete the course or were not willing to take part in the post-survey. Therefore, a total of 866 medical students completed the whole study and completed the post-survey (so the target population is 866 medical students). Male students were dominant at 55.7% of the study population, and a large number of students were from government institutions (63.5%) and were from the final year of study (57.0%).

In the case of pharmacy students, 1,195 students initially filled out the baseline data, but only 816 agreed to be part of the post-survey. Among these 816 students, 772 filled and completed the pre–post study and 44 failed to complete the course. The pharmacy student population was also dominated by male students (60.8%). A large proportion were from private institutions (63.3%) and from the final years (55.2%) (Table 1).

**TABLE 1:** Demographic details of medical and pharmacy students.

Numbers	Demographic variable	Medical students (866)		Pharmacy students (772)	
1	Gender				
	Male	482	55.7	469	60.8
	Female	384	44.3	303	39.2
2	Nature of institution				
	Private	316	36.5	489	63.3
	Government	550	63.5	283	36.7
3	Year of schooling				
	3rd	158	18.3	189	24.5
	4th	214	24.7	157	20.3
	5th	494	57.0	426	55.2
4	Approximate monthly parental income (PKR)				
	<30,000	28	3.2	142	18.4
	30,000–50000	322	37.2	166	21.5
	50,001–100000	277	32.0	34	4.4
	100,001–150000	239	27.6	430	55.7

### Pre–post evaluation of individual perception, attitude, and practice items of medical students

After comparing the median of each individual item, we saw significant changes in affinity toward PP, tactics by PCs, and gift adoption behavior of the medical students. Student views changed from “disagree” to “strongly disagree” in terms of delivering information obtained in PP to patients and that PP gives doctors confidence to counsel patients (median score 2 to 1 p < 0.001). Similarly in terms of the attitude items, a significantly high number of medical students reported that medical information should not be permitted on the website of companies (median 3 to 4 P < 0.001) and that government should mandate preapproval of all PP for prescription drugs (median 2 to 4 p < 0.001). We also saw changes in future practices and students’ behavior change from “neutral” to “disagree” in terms of prescribing antibiotics under the influence of promotional activity and prescribing antibiotics due to the influence of gifts by PCs (P < 0.001). See Table 2 for details.

**TABLE 2:** Pre-post evaluation of perception, attitude, and practice items of 886 medical students.

Numbers	Perception statements	Pre-intervention	Post-intervention	P value	Z statistics
		Median (IQR)	Median (IQR)		
1	Physicians or pharmacists have to deliver information obtained in pharmaceutical promotion about prescription drugs to patients.	2 (2)	1 (0)	<0.001	−17.99
2	Pharmaceutical promotion for prescription drugs can give doctors the confidence to talk with patients about their concerns.	2 (2)	1 (0)	<0.001	−19.81
3	Pharmaceutical promotion for prescription drugs encourages doctors to ask patients to follow treatment instructions or advice from their doctors.	2 (1)	1 (1)	<0.001	−21.41
4	Pharmaceutical promotion of drugs can improve physicians’ awareness of the medical drug or equipment.	2 (1)	3 (1)	<0.001	−15.53
5	Pharmaceutical promotion for prescription drugs interferes with the relationships between patients and doctors.	3 (2)	3 (1)	0.045	−4.35
6	Pharmaceutical promotion for prescription drugs promotes unnecessary visits to hospitals.	3 (2)	2 (1)	<0.001	−20.65
7	Pharmaceutical promotion for prescription drugs can prevent incorrect information on drugs from being spread.	3 (1)	4 (2)	<0.001	−17.29
8	Pharmaceutical promotion for prescription drugs can restrict patients’ choices for using drugs.	3 (2)	2 (1)	<0.001	−16.08
9	Pharmaceutical promotion for prescription drugs will increase the profits of pharmaceutical companies.	2 (2)	3 (0)	<0.001	−11.10
10	Pharmaceutical promotion for prescription drugs can play a role in adding the rebates of pharmaceutical companies to doctors.	3 (2)	3 (1)	<0.001	−11.58

Numbers	Attitude statements	Pre-intervention	Post-intervention	P value	Z statistics
		Median (IQR)	Median (IQR)		
1	Pharmaceutical promotion for prescription drugs is necessary for physicians.	3 (2)	3 (1)	0.173	−1.37
2	Information about drugs provided by pharmaceutical sales reps is reliable.	3 (2)	2 (1)	<0.001	−19.41
3	Are you willing to actively utilize the data obtained from pharmaceutical promotion for prescription drugs when consulting patients in the future?	3 (1)	2 (1)	<0.001	−19.56
4	Are you willing to actively accept patients’ opinions when they ask you to prescribe, fill, or administer drugs of which they have knowledge due to pharmaceutical promotion in the future?	3 (2)	2 (1)	<0.001	−17.02
5	Pharmaceutical promotion for prescription drugs should not be permitted on the websites of drug companies.	3 (2)	4 (1)	<0.001	−18.87
6	Pharmaceutical promotion for prescription drugs can create unrealistic expectations about drugs.	2 (1)	5 (1)	<0.001	−22.17
7	Pharmaceutical promotion for prescription drugs can play a part in improving patients’ drug compliance.	3 (1)	2 (2)	<0.001	−11.48
8	Pharmaceutical promotion for prescription drugs will lead to increasing drug prices due to the cost of marketing strategies.	3 (1)	4 (2)	<0.001	−8.69
9	Do you think that the government should mandate preapproval of all pharmaceutical promotion for prescription drugs if they are permitted?	2 (1)	4 (3)	<0.001	−15.04
10	It is unacceptable for a physician to receive a gift from a drug company in any form?	3 (2)	4 (4)	<0.001	−13.77
11	I would feel comfortable receiving the following gifts from a pharmaceutical company: lunch, penlight, stethoscope, textbook, and watch/jewelry.	3 (3)	3 (3)	0.621	−0.49
12	Five drugs from five different companies are identical in terms of price, efficacy and effectiveness. I would preferentially prescribe a drug from one of the companies that provided me with such gifts or incentives over those from companies that did not.	4 (2)	3 (3)	<0.001	−7.06
13	Students should not have any interaction with drug companies in medical school.	3 (2)	3 (4)	0.050	−2.78
14	The information provided about drug effectiveness from pharmaceutical companies is untrustworthy.	3 (1)	2 (2)	<0.001	−8.39
15	As long as their medications are accepted as part of standard care, it is acceptable for physicians to be compensated PKR 100 by the drug company each time their drug is prescribed.	3 (1)	1 (2)	<0.001	−17.54
16	It is acceptable for drug companies to sponsor events/educational seminars during medical school.	2 (1)	2 (2)	<0.001	−9.18
17	If a drug company agreed to pay for the printing cost of all my class notes in undergraduate medical school, I would not mind the logo of that company appearing in the bottom corner of the first slide of my presentation lecture.	3 (2)	2 (2)	<0.001	−11.13
18	Do you think these interactions should be regularized?	3 (2)	4 (2)	<0.001	−15.49
19	The gifts and other things given by pharmaceutical industries to doctors should be recorded by the government as in many developed countries.	2 (3)	4 (3)	<0.001	−5.55
20	Do you feel that there is a need for incorporating guidelines regarding the relationship between the pharmaceutical industry and medical professionals in the undergraduate curriculum?	2 (1)	4 (3)	<0.001	−6.67
21	Do you think you have been taught enough about pharmaceutical promotion handling?	3 (2)	2 (2)	<0.001	−9.59
22	Do you feel that the syllabus provides you with enough knowledge about how to interpret the knowledge given during the promotional activity?	3 (2)	2 (1)	<0.001	−11.42
23	Do you think that pharmaceutical promotion for prescription drugs can have a negative effect on physicians’ prescribing practices?	3 (2)	4 (0)	<0.001	−15.15
24	Do you feel that interactions with representatives are one of the key factors in the irrational prescribing of drugs?	3 (2)	4 (0)	<0.001	−14.69
25	Do you feel that interactions with representative are one of the key factors in the irrational prescribing of antibiotics?	3 (2)	4 (0)	<0.001	−15.36
26	Do you feel that doctors who meet representatives more often prescribe more antibiotics?	3 (2)	3 (2)	<0.001	−4.15
27	Do you feel that those doctors who accept more gifts from companies prescribe more antibiotics than others?	3 (3)	3 (3)	<0.026	−2.23
28	Is there a need to strengthen ethical standards to control the interaction between physicians and pharmaceutical companies?	2 (2)	4 (1)	<0.001	−23.61
29	Do you think that MRs should have a certificate of professional and ethical capability to execute their profession?	2 (2)	4 (1)	<0.001	−21.95

Practice statements					
1	Will you prescribe more antibiotics under the influence of promotional activity in future?	3 (2)	2 (1)	<0.001	−27.49
2	Will you prescribe more antibiotics under the influence of accepting the gifts of pharmaceutical companies?	3 (3)	2 (2)	<0.001	−27.97

5 = strongly agree, 4 = agree, 3 = neutral, 2 = disagree, 1 = strongly disagree.

### Over all pre-post knowledge, attitude and practice score of medical students

A Wilcoxon signed-rank test was used to investigate the knowledge, attitude, and practice scores between baseline and post-intervention for the group of medical students. There was a statistically significant decrease in the median (IQR) knowledge score of two points between baseline and post-data (P < 0.001). Similarly, the attitude and practice scores were also significantly reduced at P < 0.001. Details are given in Table 3. The demographic association of medical students with total perception attitude and practice score has been given in Table 4.

**TABLE 3:** Pre-post change in knowledge, attitude, and practices scores of 886 medical students.

Scores	Time points		Z-statistics	P value
	Baseline median (IQR)	Post intervention median (IQR)		
Perception	26 (9)	24 (5)	−5.67b	<0.001*
Attitude	89 (9)	71 (9)	−32.375b	<0.001*
Practices	6 (3)	2 (2)	−22.956b	<0.001*

Note: Z: Test statistics for Wilcoxon signed-rank test, IQR: Interquartile range, *Significant P value is < 0.05.

Note: Q7 for perception items were negative, so “strongly disagree” given 5 and “strongly agree” given 1 score.

Note Qs 5, 6, 8, 9, 10, 13, 14, 18, 19, 20, 23–25, 30, and 31 for attitude items were negative, so “strongly disagree” given 5 and “strongly agree” given 1 score.

**TABLE 4:** Demographic association of medical students with total perception, attitude, and practice scores.

Numbers	Demographics	Perception scores (median IQR)		Attitude scores (median IQR)		Practice score (median IQR)	
1	Gender	Pre	Post	Pre	Post	Pre	Post
	Male	28 (8)	24 (5)	88 (9)	71 (7)	7 (4)	2 (2)
	Female	24 (10)	23 (5)	89 (7)	71 (11)	6 (3)	2 (2)
	P	<0.001	0.013	0.243	0.096	0.012	0.070
2	Nature of institution						
	Private	24 (8)	24 (3)	88 (8)	69 (7)	6 (5)	2 (2)
	Government	26 (10)	24 (5)	89 (15)	71 (9)	7 (3)	2 (2)
	P	0.082	0.458	<0.001	0.001	<0.001	0.325
3	Year of schooling						
	3rd	24 (10)	22 (4)	87 (5)	74 (8)	7 (2)	2 (2)
	4th	26 (11)	24 (5)	88 (6)	71.5 (11)	6 (3)	2 (0)
	5th	26 (9)	24 (5)	90 (14)	69 (6)	7 (4)	3 (2)
	P	0.246	<0.001	0.015	<0.001	0.735	<0.001
4	Approximate monthly parental income (PKR)						
	<30,000	35 (0)	26 (13)	84 (2)	73 (15)	8 (3)	2 (0)
	30,000–50000	26 (9)	24 (5)	90 (5)	71 (10)	6 (3)	2 (2)
	50,001–100000	24 (10)	24 (5)	90 (16)	71 (8)	7 (3)	3 (2)
	100,001–150000	24 (10)	22 (2)	87 (10)	71 (7)	6 (6)	2 (2)
	P	<0.001	0.001	<0.001	0.003	0.866	<0.001

Note: Mann–Whitney and Kruskal–Wallis tests are used to evaluate the p value of the pre and post knowledge, attitude, and practice scores with demographics.

### Pre-post evaluation of individual perception, attitude and practice items of pharmacy students

After evaluating the pre and post knowledge, attitude, and practice scores of pharmacy students, we saw a significant change in them. There is a significant change from “neutral” response to “disagree” (median 3 to 2) in terms of “It is acceptable to participate in the social activities such as dinners arranged by companies” and from “agree” to “disagree” in terms of “accepting gifts for educational purposes distributed by companies, accepting drug samples given by the companies, accepting books, journals and other educational material distributed by companies” (median 4 to 2 p < 0.001). Similarly, in terms of attitude, most items got changed answers, such as from “agree” to “neutral” in terms of “Government should mandate preapproval of all PP for drugs if they are permitted” and from “disagree” to “neutral” in terms of “it is unacceptable for a pharmacist to receive a gift from a drug company in any form” (p < 0.001). Similarly, most of the students said that they would not dispense antibiotics under the pressure of gifts or any promotional influence in future. The results are mentioned in Table 5.

**TABLE 5:** Pre-post evaluation of the perception, attitude, and practice items of 772 pharmacy students.

Numbers	Perception Statements	Pre-intervention	Post-intervention	P value
		Median (IQR)	Median (IQR)	
1	Pharmacists have to deliver the information obtained in pharmaceutical promotion about prescription drugs to patients.	4 (2)	2 (1)	<0.001
2	Pharmaceutical promotion can give pharmacists the confidence to counsel patients about their concerns.	3 (1)	2 (1)	<0.001
3	The educational activities supported by companies and drug information circular provide good support for education.	3 (1)	2 (1)	<0.001
4	The promotional activities of pharmaceutical companies affect physicians’ prescribing practices.	3 (1)	3 (2)	<0.001
5	The promotional activities of pharmaceutical companies affect pharmacists’ dispensing practices.	3 (1)	2 (2)	<0.001
6	Do you think that pharmaceutical promotion can promote unnecessary visits to hospitals?	3 (1)	2 (1)	<0.001
7	Do you think that pharmaceutical promotion can prevent incorrect information on drugs from being spread?	3 (1)	3 (1)	<0.001
8	Do you think that pharmaceutical promotion can restrict pharmacists' choices when dispensing drugs?	3 (1)	3 (1)	0.91
9	Pharmaceutical promotion will increase the profits of pharmaceutical companies.	3 (1)	3 (2)	<0.001
10	It is ethical for pharmaceutical companies to finance scientific research.	3 (1)	3 (1)	0.45
11	It is acceptable to participate in social activities such as dinners arranged by companies.	3 (1)	2 (1)	<0.001
12	It is appropriate to accept gifts for educational purposes distributed by companies.	4 (1)	2 (1)	<0.001
13	I think it is appropriate to accept drug samples given by the companies.	4 (2)	2 (1)	<0.001
14	It is appropriate to accept books, journals and other educational material distributed by companies.	4 (3)	2 (1)	<0.001
15	It is appropriate to accept the support of companies to participate in congresses.	3 (2)	2 (1)	<0.001
16	Companies do not pass on promotional expenses to drug prices.	3 (2)	4 (1)	<0.001
17	Company promotions affect the advisory behavior or drug information of pharmacists	3 (1)	3 (1)	<0.001
18	Company promotions do not cause unnecessary prescribing or sales of drugs	3 (1)	4 (1)	<0.001

Numbers	Attitude statements	Pre-intervention	Post-intervention	P value
		Median (IQR)	Median (IQR)	
1	Pharmaceutical promotion for drugs is necessary for pharmacists.	4 (1)	2 (1)	<0.001
2	Are you willing to actively utilize the data obtained from pharmaceutical promotion when counseling patients in the future?	4 (1)	2 (1)	<0.001
3	Are you willing to actively accept patients’ opinions when they ask you to dispense, fill, or administer drugs pf which they have knowledge due to pharmaceutical promotion in the future?	3 (1)	2 (1)	<0.001
4	Do you think that pharmaceutical promotion should not be permitted on the websites of drug companies?	3 (1)	3 (1)	<0.04
5	Do you think that pharmaceutical promotion can create unrealistic expectations about drugs?	3 (1)	4 (1)	<0.001
6	Do you expect that pharmaceutical promotion for drugs will lead to increasing drug prices due to the cost of marketing strategies?	3 (1)	3 (1)	<0.345
7	Do you think that the government should mandate preapproval of all pharmaceutical promotion for drugs if they are permitted?	4 (1)	3 (1)	<0.001
8	It is unacceptable for a pharmacist to receive a gift from a drug company in any form.	2 (2)	3 (2)	0.03
9	Five drugs from five different companies are identical in terms of price, efficacy and effectiveness. I would preferentially dispense a drug from one of the companies that provided me with such gifts or incentives over those from companies that did not.	3 (2)	2 (0)	<0.001
10	Pharmacy students should not have any interaction with drug companies in pharmacy school.	4 (1)	2 (0)	<0.001
11	The information provided about drug effectiveness from pharmaceutical companies is untrustworthy.	3 (1)	2 (1)	<0.001
12	Do you think you are taught enough about pharmaceutical promotion handling?	3 (2)	2 (0)	<0.001
13	It is acceptable for drug companies to sponsor events/educational seminars during pharmacy school.	3 (2)	2 (0)	<0.001
14	Do you feel that the syllabus provides enough knowledge about how to interpret the knowledge given during promotional activity?	3 (2)	2 (0)	<0.001
15	Interactions between pharmacists and sales reps should be regularized.	4 (1)	5 (3)	<0.001
16	Do you think that the gifts and other things given by pharmaceutical industries to community pharmacists should be recorded by the government as in many developed countries?	4 (1)	4 (3)	<0.001
17	Do you feel that there is a need to incorporate guidelines regarding the relationship between the pharmaceutical industry and pharmacists in the undergraduate curriculum?	4 (1)	4 (3)	0.006
18	Do you think that the pharmaceutical promotion for drugs can have a negative effect on pharmacists’ dispensing practices?	3 (1)	4 (3)	<0.001
19	Do you feel that interaction with representatives is one of the key factors in the irrational dispensing of drugs?	3 (1)	4 (3)	<0.001
20	Do you feel that interaction with representative is one of the key factors in the irrational dispensing of antibiotics?	3 (1)	3 (2)	0.043
21	Do you feel that community pharmacists who meet representatives more often dispense more antibiotics?	3.5 (1)	3 (2)	<0.001
22	Do you feel that those pharmacists who accept more gifts from companies dispense more antibiotics than others?	4 (1)	4 (1)	<0.001

Practice statements				
1	Will you dispense more antibiotics under the influence of promotional activity?	3 (2)	2 (1)	<0.001
2	Will you dispense more antibiotics under the influence of accepting gifts from pharmaceutical companies?	3 (1)	2 (2)	<0.001

5 = strongly agree, 4 = agree, 3 = neutral, 2 = disagree, 1 = strongly disagree.

### Overall pre-post Knowledge attitude and practice score of pharmacy students

Table 6 demonstrates the results of the Wilcoxon signed-rank test to investigate the knowledge, attitude, and practice scores pre- and post-intervention for the group of pharmacy students. There was a statistically significant decrease in the median (IQR) knowledge score of 12 points between baseline and post data (P < 0.001). Similarly, the attitude score plunged by 11, and the practices score decreased 2 points, which was a significant reduction (P < 0.001). The demographic association between the pharmacy students and total perception, attitude and practice score has been mentioned in the Table 7.

**TABLE 6:** Pre-post change in knowledge, attitude, and practice scores of 772 pharmacy students.

Scores	Time points		Z-statistics	P value
	Baseline median (IQR)	Post intervention (IQR)		
Perception	58 (8)	47 (5)	−27.842b	< 0.001*
Attitude	67 (9)	60 (11)	−18.215b	< 0.001*
Practices	6 (3)	4 (2)	−21.156b	< 0.001*

Note: Z: Test statistics for Wilcoxon signed-rank test, IQR: Interquartile range, *Significant P value is <0.05.

Q7, 16, 18 perception items were negative, so “strongly disagree” scored 5 and “strongly agree” 1.

Q4, 10, 15, 16, 17, 18 of attitude items were negatively categorized, so “strongly disagree” scored 5 and “strongly agree” 1.

**TABLE 7:** Demographic association of pharmacy students with total perception, attitude, and practice scores.

Numbers	Demographics	Perception scores (median IQR)		Attitude scores (median IQR)		Practice score (median IQR)	
1	Gender	Pre	Post	Pre	Post	Pre	Post
	Male	59 (9)	47 (6)	68 (10)	61 (11)	6 (2)	4 (2)
	Female	57 (9)	47 (5)	66 (8)	57 (10)	6 (3)	4 (2)
	P	0.094	0.576	0.215	0.353	0.923	0.081
2	Nature of institution						
	Private	58 (9)	48 (6)	66 (8)	61 (11)	6 (2)	4 (2)
	Government	58 (7)	47 (4)	69 (10)	57 (7)	6 (3)	4 (0)
	P	0.360	<0.001	0.001	<0.001	0.526	<0.001
3	Year of schooling						
	3rd	57 (6)	45 (4)	65 (4)	66 (5)	6 (2)	4 (1)
	4th	59 (6)	47 (3)	66 (7)	61 (10)	6 (2)	4 (2)
	5th	58 (11)	49 (6)	69 (10)	55 (7)	6 (2)	4 (2)
	P	0.001	<0.001	<0.001	<0.001	<0.001	0.385
4	Approximate monthly parental income (PKR)						
	<30,000	59 (10)	47 (5)	70 (9)	55 (4)	6 (3)	4 (0)
	30,000–50000	58 (11)	47 (3)	66 (9)	61 (9)	6 (3)	4 (0)
	50,001–100000	54.5 (7)	48 (6)	68 (8)	55 (10)	6 (2)	4 (2)
	100,001–150000	58 (8)	48 (7)	66 (9)	63 (11)	6 (1)	4 (2)
	P	0.132	<0.001	<0.001	<0.001	0.555	<0.001

Note: Mann–Whitney and Kruskal–Wallis tests used to evaluate the p value of pre and post knowledge, attitude, and practice scores with demographics.

## Discussion

Students are highly affected by promotion, and it alters their perception and prescribing/dispensing behavior in the long term (Gillani et al., 2024a; Gillani et al., 2024b). This study is the first of its kind which used the WHO/HAI module for an intervention for pharmacy and medical students using an online plate form. It shows that the educational intervention proved to be effective in coping with the detrimental effects of the PP and changes students’ way of thinking about it. We conducted an online intervention due to the COVID-19 lockdown. Although the sudden shift from physical to online learning posed challenges to students, they adapted the new situation (Almendingen et al., 2021). Online interventions were very common (NIHR, 2025; Andersson, 2018; Oti and Pitt, 2021) and were seen to be effective during the COVID-19 period (Sande et al., 2023; Rodríguez-Pra et al., 2023; Fischer-Grote et al., 2024).

Our online study showed that all categories of scores and the total scores changed significantly after the module. Certain categories of scores also varied according to gender, year of schooling, nature of institution, and parental income before and after the module. The effectiveness of such physical educational programs has been proven by Shankar et al. (2012), who demonstrated that students’ views were significantly changed in many cases. Similarly, innovative sessions for medical students about PP were conducted in the USA involving two faculty members and MR to highlight physician–MR interactions (Wofford and Ohl, 2005). This study also used paper-based pre–post surveys which elicited information from participants about different aspects of the interactions. Changes in attitudes were noted after the workshop. In another survey conducted in the USA following a series of four seminars for third-year students, they were given articles on PP and asked to summarize them to other students. It was observed that students became more cautious about their relationship with PI after attending these seminars (Mar et al., 2008). Our study specifically highlighted the change in prospective assumed practices under the pressure of PP and the pressure of gifts from PCs. Students willing to prescribe or dispense antibiotics in the future due to PP change their perspective to disagreement. No evidence suggested an evaluation of such responses as in our study, but there was great improvement in the knowledge and attitude of healthcare students pre- and post-educational intervention on antibiotic use (Orok et al., 2025; Aboalshamat et al., 2019; Sayyadi-Rahaghi et al., 2023). These changes in knowledge and practices were also observed in physicians (Delsors et al., 2021; Langdridge et al., 2024). Ultimately, improvements in knowledge and attitude will lead to improved practices, with other studies showing a positive correlation between knowledge and attitude practices (Gillani et al., 2019; Lakshmi et al., 2022).

The results of both groups show that participants demonstrate less willingness to receive gifts from PCs or accept financial backing from PCs. The Nepalese study participants showed low agreement rates toward rejecting PC pens before the educational intervention, but their attitudes changed afterward. The pen/small gift bearing the PC’s name serves as a persistent reminder that connects to specific company medicines which can lead to medicine prescribing (Mintzes, 2005). Doctors should participate in a pen amnesty program which requires them to give back PCs’ pens in exchange for “no free lunch” or new pens (Yamey, 2001). As part of its PharmFree campaign, AMSA operates a pen amnesty program throughout the nation. The statement regarding MRs about untrustworthy information experiences show similar disagreement levels among both types of students. Shankar et al. (2012) demonstrated that students initially provided vague responses about unbiased information promotion but later developed a clear understanding when questioned. The WHO/HAI manual provides detailed explanations about promotional methods together with a list of trustworthy drug information sources. The low level of trust toward promotions among our cohort might stem from pharmacology practical sessions which run throughout the course (Shankar et al., 2012). The practice of meeting with MRs stands as a common marketing expense that consumes 25% of the total marketing budgets of PCs (Moghimi, 2006). Our cohort demonstrates substantial value change on two points: students should not encounter MRs, and MRs should schedule regular meetings with doctors. The information MRs provide to physicians remains helpful for medical practice where physicians can give medication samples or gifts to patients who lack financial means (Dubois, 2003). The majority of the actions of MRs focus on reaching their specified targets while intentionally leading doctors astray (Lexchin, 2012; Ziganshina et al., 2009). Medical relationships must be avoided by doctors because they violate both professional ethics and represent a waste of valuable time (Brody, 2005). Our study shows that strong guidelines and restrictive policies to be included in curricula following the module completion. A US study demonstrated that education regarding PP and stricter industry–student relationship policies led to changes in both skepticism and disapproval about such relationships within educational settings (Kao et al., 2011). Research shows that healthcare students develop their understanding and stance regarding the PI before graduation, which requires professional training programs to address MR effects beforehand (Monaghan et al., 2003).

Overall, the online module was effective in improving behaviors and proposed practices. Students wish to use independent information sources during their study. They should not, however, consider sources such as textbooks as independent medicine information sources. Therefore teaching this important skill will be more effective for tackling the poor effects of PP. There should be free access to biomedical journals through the HINARI service of WHO and every student should be made aware of how to use it.

## Conclusion

The online teaching of the WHO/HAI module was effective in changing the perception and attitudes of medical and pharmacy students about the PP. The module gave insight into small learning strategies. The incorporation of such a module in the course duration is required, and students should be made aware of and taught how to use independent sources of information regarding PP and new drugs so as to confirm the claims made by MRs.

### Practical implications of this study

By transforming evidence into policy—curriculum mandates and enhanced regulatory vigilance—our study can provide a blueprint for protecting future doctors and pharmacists from industry influence. These recommendations can be disseminated via workshops with DRAP, PPC, and pharmacy deans. Further implications follow.

#### Direct integration of the WHO/HAI module

Our results (e.g., increase in knowledge scores post-intervention; p < 0.001) support mandating this module in PharmD and medical curricula across Pakistan. We propose embedding it as a certified course in Pharmacy Ethics or Clinical Pharmacology syllabi, with annual refreshers.

#### WHO/HAI toolkit scaling

Its proven effectiveness in Pakistan supports adopting this module in low-resource regions that face aggressive pharmaceutical marketing (e.g., Bangladesh, Nigeria), adapted to local regulations.

#### Skills-based training

Improved identification of unethical promotional tactics (85% post-test accuracy) justifies adding simulation exercises (e.g., role-playing of sales rep interactions) and critical appraisal workshops for drug advertisements.

#### Strengthening DRAP’s mandate

Attitude shifts (e.g., 40% increased skepticism about sales reps’ claims) highlight the need for DRAP to collaborate with universities on consumer awareness campaigns to debunk misleading promotions.

### Strengths and limitations

This is the first study to evaluate the effect of educational intervention on medical and pharmacy student behavior regarding PP after an online module. The strength of our study was the high response rate and a large sample size. The study also had limitations. First, we used pre and then post evaluations which may have pre-test sensitivity and response shift bias (Lewis et al., 2001; Shankar et al., 2010). However, following the multiple studies and protocols, we confirmed the plausibility of our study. Second, the scoring system was developed by the authors. The nature of the questionnaire may have influenced student responses. Third, this study explores students’ involvement in PP based on questionnaire data from the students. There was no attempt to validate or verify the information provided to them, and it was not possible to inquire into the elements of the informal and hidden curriculum (HC) and how they interact with the process of changing or stabilizing students’ opinions. There is a risk that the results may under- or over-report the extent of perception and attitude toward PP. Fourth, the survey we administered looks at promotion in general and not specifically the promotion of antibiotics, but we evaluated the impact of promotion on the prescription of antibiotics. It is unlikely that promotion will affect the prescription of different classes of drugs differently. Finally, it should be noted that these findings may not be readily comparable to those of students in other countries as there may be contextual variations such as the absence of universal health insurance for all individuals.

## Data Availability

The raw data supporting the conclusions of this article will be made available by the corresponding author, without undue reservation.

## Footnotes

1
https://www.dra.gov.pk/wp-content/uploads/2022/11/Draft-Therapeutic-Goods-Advertisement-Rules-2022.pdf
